# Deformation Behavior of Inconel 625 Alloy with TPMS Structure

**DOI:** 10.3390/ma18020396

**Published:** 2025-01-16

**Authors:** Kangning Xu, Jiahui Cao, Zhiwei Zheng, Rusheng Zhao, Gaopeng Xu, Hao Wang, Jincheng Wang, Boyoung Hur, Xuezheng Yue

**Affiliations:** 1 School of Materials and Chemistry, University of Shanghai for Science and Technology, Shanghai 200082, China; 2 Department of Aerospace Engineering, Tokyo Metropolitan University, Tokyo 191-0065, Japan; 3 Department of Mechanical Engineering, The University of Melbourne, Parkville, VIC 3010, Australia; 4 School of Engineering, M050, The University of Western Australia, 35 Stirling Highway, Crawley, Perth, WA 6009, Australia; 5 Department of Metallurgical and Materials Engineering, Gyeongsang National University, Jinju-si 501, Republic of Korea

**Keywords:** Inconel 625, triply periodic minimal surface, laser powder bed fusion, compressive property, energy absorption characteristic

## Abstract

Triply periodic minimal surfaces (TPMSs) are known for their smooth, fully interconnected, and naturally porous characteristics, offering a superior alternative to traditional porous structures. These structures often suffer from stress concentration and a lack of adjustability. Using laser powder bed fusion (LPBF), we have fabricated Inconel 625 sheet-based TPMS lattice structures with four distinct topologies: Primitive, IWP, Diamond, and Gyroid. The compressive responses and energy absorption capabilities of the four lattice designs were meticulously evaluated. The discrepancies between theoretical predictions and the fabricated specimens were precisely quantified using the Archimedes’ principle for volume displacement. Subsequently, the LPBF-manufactured samples underwent uniaxial compression tests, which were complemented by numerical simulation for validation. The experimental results demonstrate that the IWP lattice consistently outperformed the other three configurations in terms of yield strength. Furthermore, when comparing energy absorption efficiencies, the IWP structures were confirmed to be more effective and closer to the ideal performance. An analysis of the deformation mechanisms shows that the IWP structure characteristically failed in a layer-by-layer manner, distinct from the other structures that exhibited significant shear banding. This distinct behavior was responsible for the higher yield strength (113.16 MPa), elastic modulus (735.76 MPa), and energy absorption capacity (9009.39 MJ/m^3^) observed in the IWP configuration. To examine the influence of porosity on structural performance, specimens with two varying porosities (70% and 80%) were selected for each of the four designs. Ultimately, the mechanical performance of Inconel 625 under compression was assessed both pre- and post-deformation to elucidate its impact on the material’s integrity.

## 1. Introduction

Porous structures are widely implemented in fields such as acoustics, thermal management, and engineering materials due to their superior attributes. These features include high porosity, increased specific surface area, improved specific strength, and the abilities for energy absorption, vibration damping, and insulation against sound and heat [1,2,3,4,5]. However, porous structures face certain challenges. Specifically, designs of these structures are prone to stress concentration at the junctions of intersecting struts, which can affect the overall structural integrity [6,7,8]. Therefore, the design and utilization of porous structures should be carefully considered for the specific environmental conditions in which they are used. The most crucial thing is that the mechanical properties of these structures are adjustable to meet the exacting demands of various engineering applications [9,10,11].

A triply periodic minimal surface (TPMS) is characterized by a topological structure with a mean curvature of zero throughout its surface [12,13]. Compared with traditional porous structures, TPMS structures offer smoothness, complete interconnectivity, and a natural porosity that mitigate the issues of stress concentration and a lack of adjustability associated with conventional designs [14]. These advantages have propelled TPMSs to the forefront of research interest. Ravichander et al. fabricated five distinct TPMS structures using 316L stainless steel and analyzed their tensile and compressive deformation behaviors [15]. The results showed that the Fischer structure outperformed in strength and energy absorption under both types of loading. In contrast, the Primitive structure showed the poorest performance under compressive and tensile conditions. Yu et al. performed a comparative study on the mechanical properties of Primitive and Gyroid structures through quasi-static compression, revealing that the Primitive structure had higher specific stiffness, strength, and energy absorption capabilities [16]. Al-Ketan et al. investigated the mechanical properties of various TPMS structures made from Maraging steel via quasi-static compression testing, with the Diamond structure showing superior mechanical performance [17]. However, Viet et al., in their study on titanium implant TPMS structures including Gyroid, Primitive, Diamond, and IWP configurations, demonstrated through compression tests and simulations that the IWP structure achieved the highest effective yield strength at a given porosity level [18]. It was noted that TPMS structures manufactured from different materials display considerable differences in performance.

As the complexity and diversity of porous structures grow, so too do the challenges faced by material shaping technologies [19,20,21]. Laser powder bed fusion (LPBF) is an additive manufacturing process that uses a high-energy laser to fully melt metal powders. This technique enables the creation of components with complex geometries through metallurgical bonding, which occurs as a result of rapid cooling [22,23,24]. LPBF operates on the premise of translating a digital 3D model into thinly sliced layers, which are then interpreted by computer-aided software. A layer of metal powder is evenly spread across a build platform, and a high-energy laser beam selectively fuses the powder based on the pattern of the current layer. This stepwise powder deposition and fusion proceed sequentially, ultimately constructing an entire part [25,26,27]. Unlike conventional subtractive manufacturing, the rapid solidification characteristic of LPBF restricts the diffusion of metal atoms and alloy elements, thus suppressing grain growth and avoiding the segregation of alloy components. As a result, LPBF-manufactured parts feature a fine-grained microstructure with a homogeneous distribution of elements, enhancing their mechanical strength and ductility [28,29,30,31]. These benefits have made LPBF a favored choice for producing intricate parts for various applications, including biomedical engineering, the automotive sector, and aerospace industries [32,33,34,35]. Inconel 625, a nickel-based superalloy, is renowned for its outstanding resistance to corrosion, fatigue, and strength at elevated temperatures. It finds extensive application in sectors such as aerospace, chemicals, and marine, where equipment is frequently exposed to severe conditions. The intricate nature of most Inconel 625 components demands sophisticated manufacturing techniques, which can incur high costs when traditional fabrication methods are employed. In light of these challenges, there is an urgent requirement to investigate the application of LPBF technology with Inconel in powder form, with the aim of reducing manufacturing costs and improving production efficiency [36,37,38,39,40]. Yadroitsev et al. determined an optimal hatch spacing of 120 μm for Inconel 625 powder within the defined parameters of the SLM process. An examination of the mechanical properties of components produced with varying strategies showed that the yield strength of Inconel 625 parts, whether fabricated transversely or longitudinally, exceeded that of their forged equivalents [41]. Leary et al. performed quasi-static uniaxial compression tests to assess the mechanical behavior of Inconel 625 lattice structures, characterizing both mechanical properties and failure modes [42]. Hu et al. conducted an experimental investigation into the mechanical behavior of Inconel 625 alloys manufactured by SLM. Tensile tests across a spectrum of temperatures indicated that SLM-produced Inconel 625 has an enhanced plastic deformability at lower temperatures [43]. Additionally, microstructural analysis highlighted the pivotal role of the inhomogeneous microstructure in intergranular cracking at higher temperatures.

In comparison with other alloys, the nickel-based alloy 625 presents unique advantages for utilization in 3D printing applications. Its key benefits encompass remarkable high-temperature performance, superior corrosion resistance, and advantageous weldability and machinability. As a result, this study elected to focus on the Nickel-625 alloy for investigation.

Lattice structures, while providing distinctive benefits and applications, also present certain potential disadvantages. Among these are the increased complexity and time investment required in design and manufacturing, difficulties in grid segmentation, and potentially low optimization efficiency. In comparison, minimal surface structures like TPMSs offer significant design adaptability. They can be customized to accommodate specific shape and functional requirements while preserving structural integrity and facilitating lightweight construction. This customization enhances the components’ strength, rigidity, and resilience. Consequently, the present paper concentrates on the mechanical characteristics of the Nickel-625 alloy integrated with a TPMS structure.

In this study, the design models chosen within the TPMS framework were the Gyroid, Primitive, Diamond, and IWP structures. LPBF was employed to manufacture parts from Inconel 625. An exhaustive examination and discourse were undertaken on the compressive behavior and energy absorption features of these four TPMS configurations across a spectrum of porosity levels. Furthermore, finite element analysis (FEA) was harmonized with experimental compression studies to scrutinize the stress distribution across the distinct structural designs. The research systematically investigated the interrelation between the type of porous architecture, the alloy’s microstructural attributes, and its mechanical performance through a combined analytical and empirical approach. This encompassed an assessment of microstructural evolution and structural responses to deformation. The study delineates the interconnection between structure and properties in porous, high-temperature nickel-based superalloys.

## 2. Materials and Methods

### 2.1. Design of the TPMS Lattices

**Figure 1:**
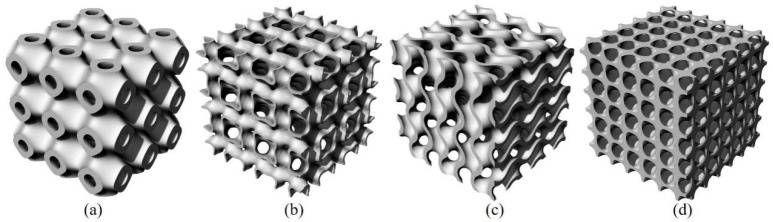
The design of TPMS structures: (**a**) Primitive, (**b**) IWP, (**c**) Gyroid, and (**d**) Diamond.

Specimens of Diamond, Gyroid, IWP, and Primitive structures with relative densities of 20% and 30% were meticulously fabricated using the open-source TPMS generator MSLattice. To assess their mechanical properties, cubic samples measuring 10 mm × 10 mm × 10 mm were created and underwent compression testing, as illustrated in Figure 1. The mathematical formulations representing the structural designs for the Primitive (Equation (1)), IWP (Equation (2)), Gyroid (Equation (3)), and Diamond (Equation (4)) configurations are presented as follows [44]:

(1) $$F_{Primitive}=cos2\pi x+cos2\pi y+cos2\pi z+C$$

(2) $$F_{IWP}=cos2\pi xcos2\pi y+cos2\pi ycos2\pi z+cos2\pi zcos2\pi x+C$$

(3) $$F_{Gyroid}=cos2\pi xsin2\pi y+cos2\pi ysin2\pi z+cos2\pi zsin2\pi x+C$$

(4) $$F_{Diamond}=sin2\pi xsin2\pi ysin2\pi z+sin2\pi xcos2\pi ycos2\pi z+cos2\pi xsin2\pi ycos2\pi z+cos2\pi xcos2\pi ysin2\pi z+C$$

where *C* is the parameter that controls the surface offset.

**Figure 2:**
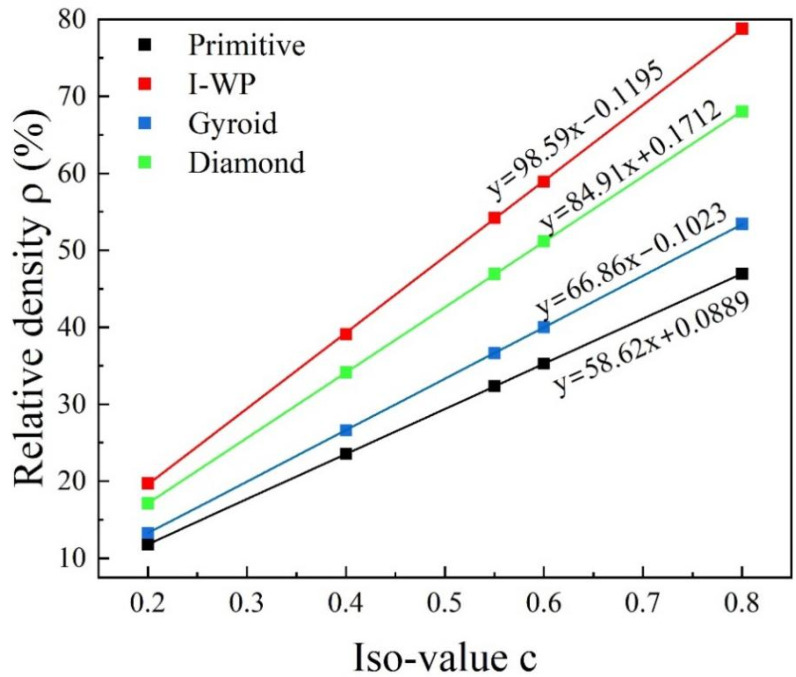
The relation between the value of the equivalent parameter *C* and the relative density of the structure.

The described method yields a TPMS structure without thickness. To engineer a sheet-like TPMS with a specific thickness, adjusting the parameter *C* to control the spatial orientation of the surface is crucial. This fine-tuning allows the TPMS to effectively divide space, thus establishing the desired sheet-like TPMS configuration. Figure 2 illustrates the relationship between the parameter *C* and the relative density of the resulting porous structure.

### 2.2. LPBF Manufacturing Process

Employing LPBF technology, Inconel 625 alloy particles were produced via a gas atomization process, resulting in a spherical particle size distribution of 35 ± 6 μm. The chemical composition of the Inconel 625 alloy is outlined in Table 1. The LPBF process was executed on an EOS M280 machine, produced by EOS GmbH, Krailling, Germany. The operational parameters for the procedure included a laser power of 285 W, a scanning speed of 960 mm/s, a spot diameter of 0.11 mm, and a layer thickness of 50 μm. The fabrication process was carried out under an inert atmosphere, utilizing argon gas as the protective environment. To alleviate the thermal stress caused by the rapid cooling rates inherent to the LPBF process, the fabricated samples were subjected to an annealing heat treatment. This treatment took place in a high-temperature vacuum furnace, where the samples were held at a temperature of 630 °C for a period of 10 h, followed by cooling within the furnace.

**Figure 3:**
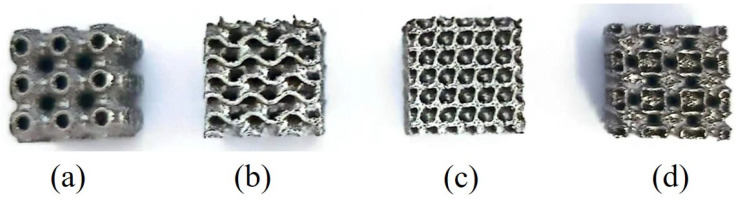
The as-fabricated TPMS structures: (**a**) Primitive, (**b**) IWP, (**c**) Gyroid, and (**d**) Diamond.

**Figure 4:**
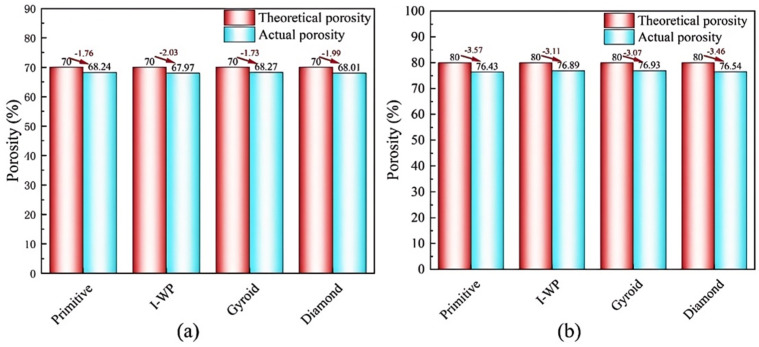
The error of theoretical porosity versus actual porosity: (**a**) 70% porosity and (**b**) 80% porosity.

The lattice structure of Inconel 625 with dimensions of 10 × 10 × 10 mm^3^, produced by LPBF, is shown in Figure 3. It should be noted that there was a discrepancy between the nominal dimensions of the designed part and the actual dimensions of the LPBF-manufactured samples. This discrepancy is attributed to two main factors: first, the adherence of metal powder particles to the sample’s exterior during the LPBF process, which caused an increase in both the length and width dimensions of the lattice structure; second, the cutting process on the substrate, which led to material loss along the cutting plane. Therefore, the dimensional deviation in the LPBF-produced samples is a critical consideration. The porosity of these samples was measured using the Archimedes drainage method, and Figure 4 displays the error plot comparing the designed porosity with the actual porosity of the specimens. The actual porosities of the fabricated samples were observed to be lower than the theoretical values predicted by their three-dimensional digital models, with a more significant discrepancy at 80% porosity than at 70%. Specifically, at 70% porosity, the discrepancy ranged from 1.73% to 2.03%. However, at 80% porosity, the discrepancy extended to 3.07% to 3.57%. This increased discrepancy was due to the adherence of powder particles to the sample surface during fabrication process, affecting the length and width dimensions. After fabrication, samples were removed from the substrate using a wire cutting machine, which resulted in a loss of material in the height dimension. Additionally, as porosity increases, the complexity of fabricating thin-walled structures grows, further widening the gap between actual and theoretical porosity values. Nonetheless, it is important to highlight that the four TPMS structures manufactured via LPBF demonstrated a high level of forming accuracy, with their porosity discrepancies considered acceptable and manageable.

### 2.3. Heat Treatment Prior to the Experiment

As a method of heat treatment, annealing serves to enhance the interlayer bonding strength in components printed via LPBF, thereby improving the mechanical properties of the entire material and reducing the porosity within the printed parts.

**Figure 5:**
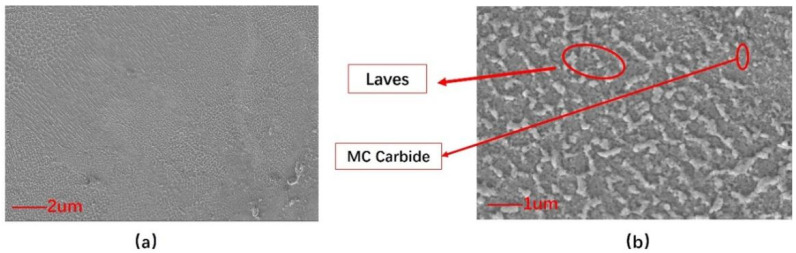
SEM image after 10 h incubation at 950 °C. (**a**) SEM images with a scale bar of 2 μm; (**b**) SEM images at a scale of 1 μm.

Figure 5, obtained after a 10 h annealing treatment at 950 °C, presents the microstructural examination of the sample through scanning electron microscopy (SEM). The resulting longitudinal section displays the expected dendritic microstructure, with the SEM micrograph highlighting the presence of both large, irregularly shaped, white precipitates and a uniform distribution of fine nanoscale particles throughout the dendritic areas. The subtle refinement of the dendritic pattern at this annealing temperature, as well as the less defined recrystallized grain boundaries evident in Figure 5b, points to the beginning of recrystallization within the microstructure. Furthermore, the interdendritic regions are marked by the presence of irregularly shaped Laves phase regions and a sparse distribution of nanoscale MC-type carbide particles.

### 2.4. Compression Tests

The Zwick/Roell Z100 universal testing machine, manufactured by Zwick/Roell in Ulm, Germany, was utilized for quasi-static compression testing. In accordance with the GB/T 31930-2015 standard [45], the testing was conducted at a strain rate of 0.001 s^−1^, equivalent to approximately 0.03 mm/s. The quasi-static uniaxial compression tests were oriented with the compression direction aligned parallel to the build direction of the prints, with all tests taking place at ambient temperature. The test concluded upon achieving a 50% deformation in the sample or reaching a load of 48 KN. The deformation behavior and corresponding failure modes were documented using a video camera, while the load–displacement curves were simultaneously recorded. Compressive stress (*σ*) was determined by dividing the compressive force by the sample’s apparent cross-sectional area, and compressive strain (*ε*) was calculated by dividing the displacement by the initial height of the sample.

### 2.5. Phase Characterization

To ascertain the structural organization and phase composition, an X-ray diffraction (XRD) analyzer was employed to examine the phases present within the components. The scan was conducted over a 2θ range from 20° to 90° at a rate of 5° per minute. The acquired data were subsequently imported into MDI Jade6 software for comparison with standard PDF cards, thereby determining the phase composition of the specimen. The XRD equipment utilized was the Bruker D8 Advance.

### 2.6. Finite Element of the Mechanical Response

To achieve a more profound understanding of the stress distribution and deformation behavior within the four TPMS structures under compressive loading, finite element simulations of the compression process were conducted for each TPMS mesh using Abaqus/Explicit 2016. Recognizing potential discrepancies between the LPBF-fabricated samples and the original CAD models, the finite element analyses presented herein were aimed at qualitatively evaluating the deformation process and the characteristics of stress distribution for each structure. As a result, the original CAD models were utilized for the finite element analysis. The analytical procedure included several key steps: specifying the material properties, generating the structural mesh, applying appropriate loads and boundary conditions, and, finally, calculating the outcomes.

1. Material properties were defined by fabricating tensile specimens of Inconel 625 using the LPBF process with consistent laser processing parameters. Subsequently, the tensile properties of these specimens were evaluated using a universal testing machine. The resultant mechanical property parameters, including Poisson’s ratio, tensile strength, and modulus of elasticity, were obtained and are detailed in Table 2.

2. For structural meshing within the simulation, discrete rigid elements from the R3D4 element type were utilized for the two compression plates, while solid elements from the C3D10M element type were employed for the meshes.

3. Load and boundary condition application: Reflecting the actual compression process, where the maximum compression displacement is 5 mm, the finite element analysis accordingly imposed a 5 mm displacement as the boundary condition. Additionally, the lower surface of the structure was assigned as a fixed support.

4. Results calculation: following the definition of material properties, structural meshing, and the application of loads and boundary conditions, the finite element analysis proceeded to solve for the structural deformation and the stress distribution state during the compression process.

## 3. Results

### 3.1. Formability and Microstructure

**Figure 6:**
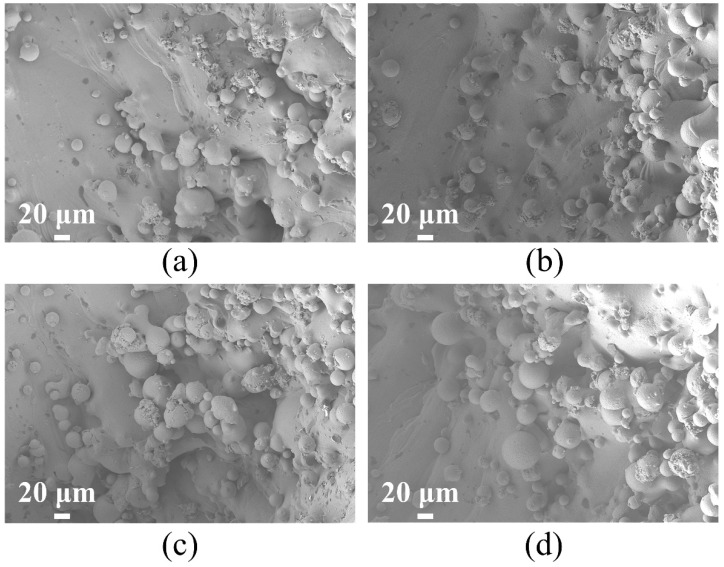
SEM images of four TPMS structures: (**a**) Primitive, (**b**) IWP, (**c**) Gyroid, and (**d**) Diamond.

To reduce the influence of fabrication process defects on the mechanical properties of the structures, the four TPMS configurations were manufactured using refined process parameters. The quality of the structures, which were produced under different LPBF conditions, was comparable and is detailed in Table 3. Taking the Primitive structure as an example, the corresponding microscopic images are depicted in Figure 6. Light microscopy observations indicated the presence of powder adhesion at the structural peripheries. This can be ascribed to the staircase effect of the LPBF process, wherein a high-energy laser creates a molten pool. The rapid melting of metal powder by the laser leads to the formation of bubbles within the melt pool due to entrapped gases during the solidification phase. The quick cooling rate does not allow the molten material to fully fill these bubbles, resulting in void formation. Furthermore, the phenomenon of powder bonding is a notable aspect of the laser process, occurring at the melt pool’s edge where the laser-induced fusion of the melted powder with the adjacent unmelted powder takes place. During solidification, the partially molten or unmelted powder at the melt pool’s boundary adheres to the component’s surface.

**Figure 7:**
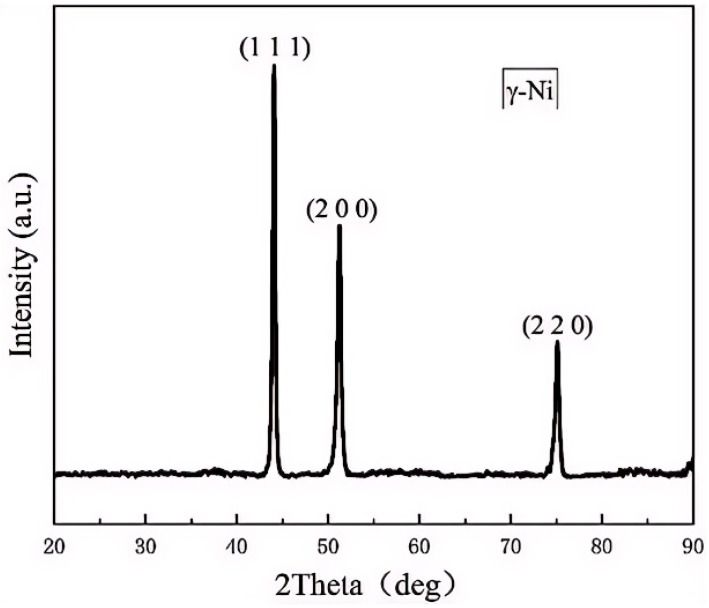
The XRD diffraction curves of Inconel 625 parts made by LPBF.

In order to determine whether phase transformation occurs in the Inconel 625 alloy during the LPBF manufacturing process and further affects its mechanical properties, XRD analysis was conducted, with the results illustrated in Figure 7. The Inconel 625 samples displayed prominent diffraction peaks at 2θ angles of 43.883°, 50.716°, and 75.289°. The analysis, facilitated by the PDF database in Jade software, revealed that these diffraction patterns are consistent with those of pure nickel. This observation suggests that the predominant phase in the specimen was an FCC structure, with the majority of the material existing in a γ-Ni solid solution state. The presence of a significant number of solute atoms such as Cr, Mo, and Nb, which are solidly dissolved in the Ni matrix, was confirmed. Notably, the XRD patterns did not indicate any intermetallic compounds or nano-scale precipitate phases. However, the absence of other phases in the nickel-based specimens could have been due to the limitations of XRD in detecting phase compositions that are less than 5% of the total material.

### 3.2. Mechanical Properties and Energy Absorption of TPMS Structures

**Figure 8:**
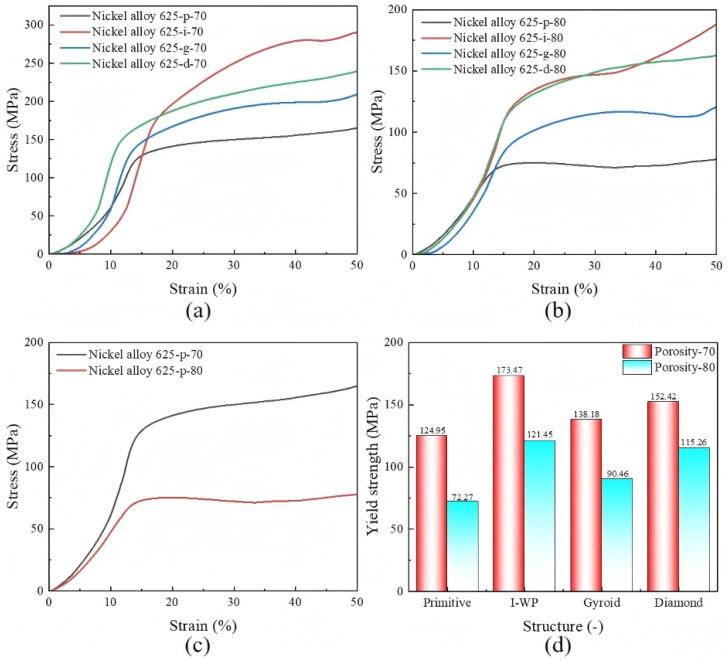
Stress–strain distribution of different TPMS structures: (**a**) 70% porosity, (**b**) 80% porosity, (**c**) comparison of Primitive structures with different porosities, and (**d**) yield strength of different TPMS structures.

**Figure 9:**
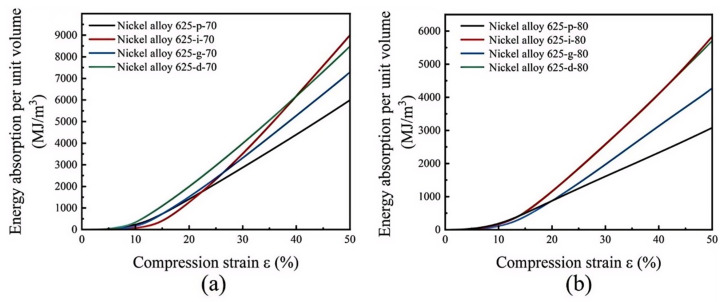
The energy absorption curves for four TPMS structures: (**a**) 70% porosity and (**b**) 80% porosity.

To assess the compressive properties of the four LPBF-processed TPMS structures, room-temperature uniaxial compression tests were performed. The resulting stress–strain curves for each distinct TPMS structure are depicted in Figure 8. These curves illustrate the three distinct phases of compressive behavior as outlined by Ashby: the linear elastic, yield, and densification phases. As shown in Figure 8a and Figure 9b, the initial linear elastic phase was observed for all TPMS structures. Upon reaching peak stress, each structure entered a plastic deformation phase characterized by an initial decrease in stress due to the buckling of the structural walls, followed by an increase as additional layers engage. This resulted in stress fluctuations during the plastic deformation phase, with a notable rise upon entering the densification phase, which is attributed to self-contact within the structure’s internal elements. Figure 8c provides a comparative analysis of the stress–strain curves for Primitive structures with 70% and 80% porosities, showing a 10% porosity variation corresponds to a roughly 40 MPa difference in their maximum yield strength. Figure 8d summarizes the maximum yield strengths across the different TPMS structures, with the IWP structure exhibiting the highest values of 173.47 MPa and 121.45 MPa at 70% and 80% porosities, respectively. This was followed by the Diamond structure at 152.42 MPa and 115.26 MPa, the Gyroid structure at 138.18 MPa and 90.46 MPa, and the Primitive structure at 124.95 MPa and 72.27 MPa.

The capacity of a porous material to absorb energy is a critical parameter in determining its suitability for applications in energy-absorbing devices. The energy absorption per unit volume of a porous material is calculated as follows [46]:

(5) $$W_{v}=\int_{0}^{\varepsilon_{d}}\sigma (\varepsilon )d\varepsilon$$

where *W_ν_* is the cumulative energy absorption per unit volume of porous material in MJ/m^3^, *ε* is the strain, *σ*(*ε*) is the compressive stress corresponding to strain *ε* in MPa, and *ε_d_* is the upper limit of compressive strain.

The integration of the stress–strain curves delineates the relationship between the absorbed energy *W_ν_* per unit volume and the compressive strain for the various TPMS structures, as illustrated in Figure 9. The observations from the figure reveal that the IWP structure surpassed the other three structures in energy absorption capacity, regardless of whether the porosity was 70% or 80%. However, it is observed that the Diamond structure initially displayed a higher energy absorption curve compared with the IWP structure at lower strains. As the strain increased, the load-bearing capacity of the Diamond structure decreased, causing its energy absorption curve to progressively fall below that of the IWP structure. At a strain of 50%, the cumulative energy absorption per unit volume for the IWP structure with 70% porosity was 9009.39 MJ/m^3^; for the Diamond structure, it was 8471.05 MJ/m^3^; for the Gyroid structure, it was 7237.69 MJ/m^3^; and for the Primitive structure, it was 5973.74 MJ/m^3^. These results indicate that the IWP structure had the highest energy absorption capacity among the evaluated structures.

For a thorough assessment of the mechanical properties of a TPMS structure, especially in relation to its structural characteristics, an in-depth analysis of its stress–strain behavior is imperative. The modulus of elasticity (E) for a porous structure can be determined by calculating the slope of the linear region within the initial elastic phase of the stress–strain curve. The yield strength (*σ_m_*) is identified as the stress value at the intersection where a line parallel to the initial linear elastic segment, offset by 0.2% strain, crosses the stress–strain curve. The platform stress (*σ_p1_*) is obtained by averaging the stress values within the 20% to 40% strain range. The computational findings are presented in Table 4, which indicate that the IWP structure exhibited a higher modulus of elasticity, yield strength, and platform stress compared with the other three structures. This further underscores the superior mechanical properties of the IWP structure among the four evaluated TPMS structures.

**Figure 10:**
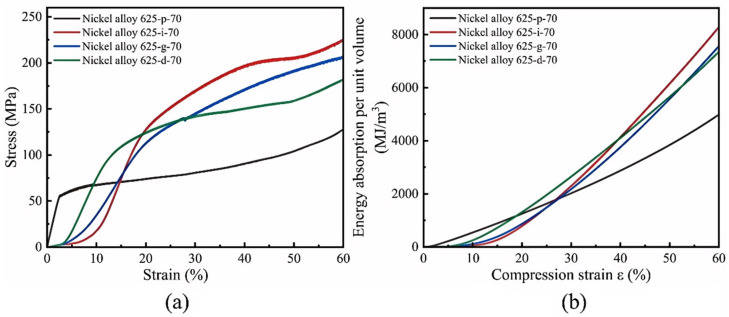
(**a**) Stress–strain distribution of different TPMS structures with 70% porosity at 250 °C; (**b**) the energy absorption curves for four TPMS structures with 70% porosity at 250 °C.

Supplementing the analysis, high-temperature compression tests were also performed on the four TPMS structures fabricated via LPBF, with the corresponding stress–strain curves displayed in Figure 10a. It is particularly significant that the IWP structure, even at a 70% porosity level, still achieved the highest yield strength, peaking at 225.76 MPa. The integration of these curves reveals the relationship between the absorbed energy per unit volume and the compressive strain for the different TPMS structures, as shown in Figure 10b. The graph distinctly illustrates the IWP structure’s superior performance in energy absorption capacity. At a strain of 50%, the cumulative energy absorption per unit volume for the IWP structure at 70% porosity was recorded at 8284.58 MJ/m^3^, contrasting with 7350.49 MJ/m^3^ for the Diamond structure, 7563.18 MJ/m^3^ for the Gyroid structure, and 4987.16 MJ/m^3^ for the Primitive structure. These results suggest that under high-temperature compression testing, the IWP structure maintains the highest yield strength and the most advantageous energy absorption capacity, consistent with the findings from room-temperature compression tests and further validating the IWP structure’s superior attributes.

### 3.3. Deformation Behaviors of TPMS Structures

**Figure 11:**
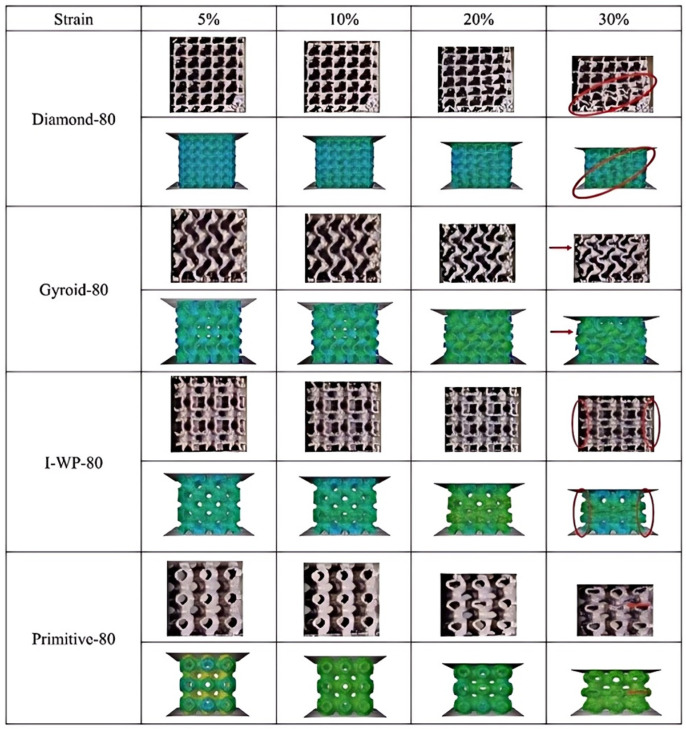
Comparison of compression experiments and stress clouds of four TPMS structures at different strains.

During compression, a structure experiences incremental deformation with escalating strain, leading to fracture and the loss of load-bearing capacity upon reaching a critical strain threshold. Figure 11 depicts the compressive deformation behavior of the four TPMS structures with 80% porosity under quasi-static uniaxial compression at strains of 5%, 10%, 20%, and 30%, along with the corresponding stress distributions at these strain levels. For the Gyroid, IWP, and Primitive structures, deformation was not apparent until the strain reached 20%, at which point these structures began to show lateral expansion at their peripheries and layer-by-layer plastic deformation. This transverse expansion was especially noticeable at a strain of 30%, suggesting a deformation mechanism characterized by continuous hardening without an accompanying change in stress. In contrast, the Diamond structure exhibited significant deformation only when the strain exceeded 20%. Upon further strain increase to 30%, the Diamond structure underwent severe damage, with plastic deformation predominantly in the diagonal direction, indicating that the 45° shear zone is the primary deformation mechanism for this structure. However, the stress–strain curve for the Diamond structure did not display significant fluctuations. It is noted that stress fluctuations are absent in lattice structures above a certain critical relative density [15]. Given the high porosity of all lattice structures in this study, they demonstrated a continuous hardening response during compressive loading.

The stress distributions for the four structures were simulated using FEA. A comparison between the FEA predictions and the actual compression observations was made, with red arrows and circled areas highlighting points of agreement between the simulation and experimental reality. For the Diamond structure, compressive stresses were predominantly localized along the structure’s diagonals, with the maximum stress observed at 2614 MPa. This stress distribution pattern resulted in preferential fracturing at the points of concentration during compression, specifically along the diagonals, which was confirmed by the actual compression process. The structure initially fractured at the diagonal positions in exact accordance with the simulated results.

In the case of the Gyroid structure, the stress distribution prior to fracture indicates a shift in the location of stress concentration due to architectural features. Stresses were predominantly concentrated on the protruding curved surfaces, reaching a maximum of 2599 MPa. This led to fracturing initiating at these areas of stress concentration, followed by transverse expansion and a layer-by-layer collapse mechanism under compression, consistent with both the actual compression process and the simulated outcomes.

For the IWP structure, the stress distribution diagram before collapse shows that, as a hollow and thin-walled structure, stress concentration was primarily in the middle two layers, peaking at 2523 MPa. This resulted in preferential collapse in the third layer, followed by the second, and lateral expansion due to compression, forming a layer-by-layer collapse mechanism. This was affirmed by both the actual compression process and the simulation, confirming that the collapse initiated in the middle layers.

Regarding the Primitive structure, the stress distribution cloud diagram before collapse reveals that, similar to the IWP structure, the stress concentration was chiefly in the middle layer, with a maximum stress of 1345 MPa. Unlike the IWP structure, which had multiple layers, the Primitive structure, with only three layers, showed preferential collapse in the middle layer, with a significant deformation of the central monoclonal cell. The actual compression process reflected the simulation, with collapse and severe deformation occurring in the middle layer.

## 4. Discussion

**Figure 12:**
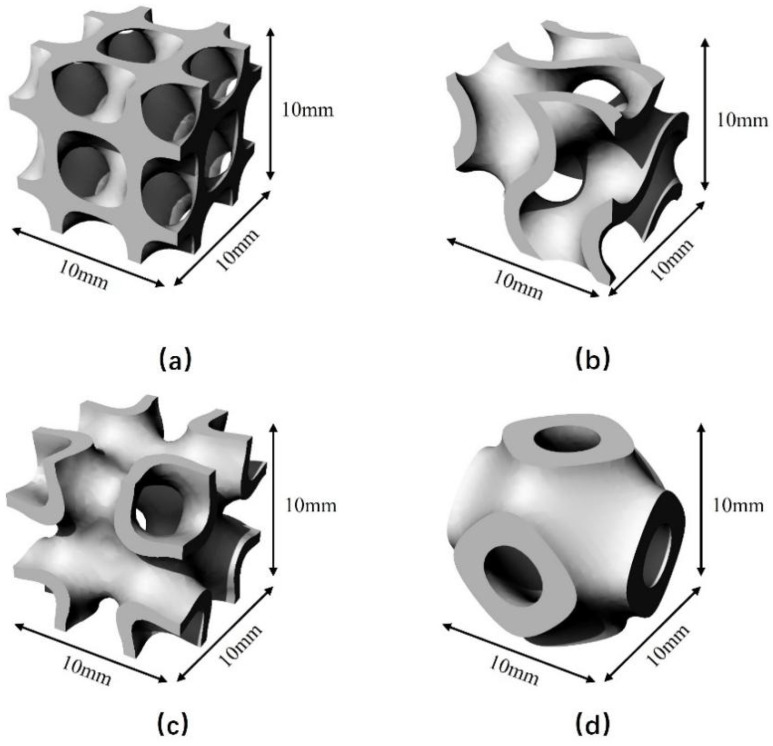
Cells of four TPMS structures. (**a**) The cell of D-TPMS, (**b**) the cell of G-TPMS, (**c**) the cell of IWP-TPMS, and (**d**) the cell of P-TPMS.

Indeed, the yield strength of these four structures is closely related to their cell structure. The cell structure of the four structures is shown in Figure 12. The cell of the P-TPMS is similar to spheroids, the cells of the D-TPMS and IWP-TPMS are mainly cubes, and the cell of the G-TPMS is mainly an S-shaped arc structure. The compressive resistance of these three shapes is in the order of sphere < S-shaped arc shape < cube. Therefore, the yield strength of the P-TPMS structure is the smallest, followed by the G-TPMS structure. However, because the final deformation of the D-TPMS structure is a 45° shear zone, the IWP-TPMS structure presents a deformation mechanism of layer-by-layer collapse, so the stability of the IWP-TPMS structure is better, and its yield strength is also the largest. Therefore, it can be explained that among the four structures, the IWP-TPMS has the best mechanical properties and P-TPMS structures have the worst mechanical properties.

In fact, the yield strength of these four structures is closely related to their cell structures. Figure 12 shows the single-cell diagrams of the four structures. The differences in the mechanical properties of the four structures are mainly as follows.

Firstly, in terms of shape, it can be seen from the figures that the single-cell structure of the P-TPMS is spherical, while the single-cell structures of the D-TPMS and IWP-TPMS are mainly cubic, and the single-cell structure of the G-TPMS is mainly arc-shaped. When compressing the four structures with the same volume, the stress area at the top of these three shapes is ranked as body < arc-shaped body < cube, and the compressive strength of these three shapes is ranked as sphere < S-shaped arc-shaped body < cube. This explains why the yield strength of the P-TPMS structure is the lowest.

Secondly, the G-TPMS structure is composed of curved surfaces, while the D-TPMS and IWP-TPMS structures are supported by pillars. When compressed, the more easily bent curved surface structure of the G-TPMS is more likely to bend, so its yield strength is lower than that of the D-TPMS and IWP-TPMS.

However, for the D-TPMS and IWP-TPMS structures, both are supported by pillars. Therefore, it is difficult to explain why the yield strength of the IWP-TPMS structure is higher than that of the D-TPMS structure based on shape alone. However, from the perspective of deformation mechanism, the D-TPMS structure eventually deforms into a 45° shear band, while the IWP-TPMS structure collapses during deformation. Since the D-TPMS produces a 45° shear band, the stress will be dispersed and offset, while the stress of the IWP-TPMS structure is always perpendicular to the plane downward. This explains why the mechanical performance of the IWP-TPMS structure is optimal. Therefore, the yield strength of the four structures is ranked as P-TPMS < G-TPMS < D-TPMS < IWP-TPMS.

## 5. Conclusions

In this study, four variations of TPMS porous materials, specifically Primitive, IWP, Diamond, and Gyroid, with porosities of 80% and 70% were modeled in Section 2 utilizing the LPBF technique. Inconel 625 was employed as the base material for fabricating the corresponding samples. The quality assessment of the molded parts included an examination of surface topography and a comparison of the original CAD models with the actual manufactured models. The mechanical properties, deformation mechanisms, and energy absorption capacities of the specimens, with different porosities and TPMS structures, were investigated through a synergistic approach of quasi-static uniaxial compression testing and numerical simulations. The key findings from this study are summarized as follows:

- A minor adherence of powder particles to the surface of the LPBF-fabricated samples, utilizing Inconel 625 as the base material, was observed in the Primitive, IWP, Diamond, and Gyroid structures. This resulted in an increase in the weight and surface roughness of the samples, as well as a reduction in the actual measured porosity. Nevertheless, the presence of these particles did not notably impact the overall fabrication quality of the four structures.
- The mechanical properties of the four TPMS structures under compressive loading are intricately linked to their structural variations. Among these structures, the IWP exhibited the highest yield strength, reaching 113.16 MPa, and also possessed the greatest energy absorption capacity, recorded at 9009.39 MJ/m^3^.
- The finite element simulation outcomes indicate that the hollow, non-suspended features of the IWP structure markedly influence the stress distribution and demonstrate superior compressive capabilities. Furthermore, the locations of structural collapse predicted by the finite element analyses were in concordance with the experimental observations.

## Figures and Tables

**Table 1:** Chemical composition of Inconel 625 alloy.

Element	Min (Wt%)	Max (Wt %)
Ni	Bal.	58.00
Cr	20.00	23.00
Mo	8.00	10.00
Nb	3.15	4.15
Fe	-	5.00
Ti	-	0.40
Al	-	0.40
Co	-	1.00
Si	-	0.50

**Table 2:** The mechanical parameters of Inconel 625 solid parts fabricated by LPBF.

Poisson’s Ratio (mm/mm)	Tensile Strength (MPa)	Young Modulus (GPa)
0.33	609.6	205

**Table 3:** The mass of LPBF-built Inconel 625 TPMS structures.

Relative Density	Primitive	IWP	Gyroid	Diamond
70	2.681 g	2.703 g	2.678 g	2.700 g
80	1.983 g	1.950 g	1.947 g	1.980 g

**Table 4:** Mechanical properties of four TPMS structures with 80% porosity.

Structure	*E* (MPa)	*σ_m_* (MPa)	*σ_p1_* (MPa)
Diamond	729.86	112.252	145.63
Gyroid	560.18	86.16	111.48
IWP	735.76	113.16	147.54
Primitive	501.82	71.27	72.84

## Data Availability

The original contributions presented in the study are included in the article, further inquiries can be directed to the corresponding author.
